# Transcription factor control of growth rate dependent genes in *Saccharomyces cerevisiae*: A three factor design

**DOI:** 10.1186/1471-2164-9-341

**Published:** 2008-07-18

**Authors:** Alessandro Fazio, Michael C Jewett, Pascale Daran-Lapujade, Roberta Mustacchi, Renata Usaite, Jack T Pronk, Christopher T Workman, Jens Nielsen

**Affiliations:** 1 Center for Microbial Biotechnology, Department of Systems Biology, Technical University of Denmark, Building 223, DK-2800, Kgs. Lyngby, Denmark; 2 Center for Biological Sequence Analysis, Department of Systems Biology, Technical University of Denmark, Building 208, DK-2800 Kgs. Lyngby, Denmark; 3 Kluyver Centre for Genomics of Industrial Fermentation and Department of Biotechnology, Delft University of Technology, Julianalaan 67, 2628 BC, Delft, The Netherlands; 4 Department of Genetics, Harvard Medical School, Boston, MA 02115, USA; 5 Department of Chemical and Biological Engineering, Chalmers University of Technology, SE- 412 96, Gothenburg, Sweden

## Abstract

**Background:**

Characterization of cellular growth is central to understanding living systems. Here, we applied a three-factor design to study the relationship between specific growth rate and genome-wide gene expression in 36 steady-state chemostat cultures of *Saccharomyces cerevisiae*. The three factors we considered were specific growth rate, nutrient limitation, and oxygen availability.

**Results:**

We identified 268 growth rate dependent genes, independent of nutrient limitation and oxygen availability. The transcriptional response was used to identify key areas in metabolism around which mRNA expression changes are significantly associated. Among key metabolic pathways, this analysis revealed *de novo *synthesis of pyrimidine ribonucleotides and ATP producing and consuming reactions at fast cellular growth. By scoring the significance of overlap between growth rate dependent genes and known transcription factor target sets, transcription factors that coordinate balanced growth were also identified. Our analysis shows that Fhl1, Rap1, and Sfp1, regulating protein biosynthesis, have significantly enriched target sets for genes up-regulated with increasing growth rate. Cell cycle regulators, such as Ace2 and Swi6, and stress response regulators, such as Yap1, were also shown to have significantly enriched target sets.

**Conclusion:**

Our work, which is the first genome-wide gene expression study to investigate specific growth rate and consider the impact of oxygen availability, provides a more conservative estimate of growth rate dependent genes than previously reported. We also provide a global view of how a small set of transcription factors, 13 in total, contribute to control of cellular growth rate. We anticipate that multi-factorial designs will play an increasing role in elucidating cellular regulation.

## Background

Regulation of cell growth is of crucial importance for the survival of all living cells. Much effort, therefore, has focused on understanding the mechanisms that control how cells achieve balanced growth, e.g. control of the cell cycle and biosynthesis of cellular building blocks. To date, DNA microarray technology [1,2] has had a considerable impact in defining causal relationships between different growth conditions and the transcriptional response of cells. A number of previous studies in *S. cerevisiae *have focused on the genome-wide transcriptional response of cells to nutrient limitation [3-5], oxygen availability [6-8] and growth rate (Table 1).

**Table 1:** Studies of growth rate effect on transcriptional response in *Saccharomyces cerevisiae*

*Study*	*Strain*	*Cultivation Mode*	*Limiting Nutrient*^*a*^	*O*_2_*Availability*	*D (h*^-1^*)*	*Array type*
Hayes *et al*. (2002)	FY1679^b^	Batch/Chemostat	C/N	Aerobic	0.1–0.2	Membrane/Glass slide
Pir *et al*. (2006)	BY4743^c^	Chemostat	C/N	Aerobic	0.1–0.2	Affymetrix Yeast S98 GeneChip
Regenberg *et al*. (2006)	CEN.PK113-7D^d^	Chemostat	C	Aerobic	0.02-0.05-0.1-0.2-0.25-0.33	Affymetrix Yeast S98 GeneChip
Castrillo *et al*. (2007)	FY1679^b^	Chemostat	C/N/P/S	Aerobic	0.07-0.1-0.2	Affymetrix Yeast S98 GeneChip
Brauer *et al*. (2008)	DBY10085^d ^DBY9492^d ^DBY9497^d^	Chemostat	C/N/P/S/U/L	Aerobic	0.05-0.1-0.15-0.2-0.25-0.3	Agilent Yeast V2 (Cy3/Cy5)
This Study	CEN.PK113-7D^d^	Chemostat	C/N	Aerobic/Anaerobic	0.03-0.1-0.2	Affymetrix Yeast S98 GeneChip

^a ^C, carbon; N, nitrogen; P, phosphorus; S, sulfur; U, uracil; L, leucine

^b ^Isogenic to S288C

^c ^S288C-derived

^d ^CEN.PK-derived

To identify growth rate dependent genes, two major requirements must be met. First, the specific growth rate of the culture (h^-1^) must be controlled. This is necessary to eliminate variability that is inherent in dynamic batch cultivation [7,9-11]. The general approach for obtaining constant specific growth rate is through continuous i.e. chemostat cultivation. Here the specific growth rate is kept constant by continuously feeding a culture with fresh nutrients having one limiting reagent at a specific dilution rate (*D*). The dilution rate is adjusted to obtain different specific growth rates. Second, it is also important to measure gene expression patterns over a range of specific growth rates. By studying factors in addition to specific growth rate (e.g. nutrient limitation), growth rate dependent genes that are independent of environmental factors can be identified.

Previous works have suggested that growth rate has a tremendous influence on the yeast transcriptional program. Specifically, Regenberg *et al*. [12] described more than 2400 growth rate dependent genes and proposed a role for the chromosomal location in the regulation of these genes. Castrillo *et al*. [13] adopted a systems biology approach to investigate the effect of growth rate at the transcriptome, proteome and metabolome levels. They identified about 900 genes whose expression is growth regulated and concentrated, in particular, on the role of the TOR complex 1. More recently, Brauer *et al*. [14] determined that transcript levels of more than one quarter of all yeast genes are linearly correlated with growth rate. While growth rate dependent genes have been identified from single factor studies [12] and two factor designs, such as growth rate and nutrient limitation [13,14], multi-factor designs, such as the approach presented here, are expected to identify growth rate dependent genes that are more independent of the specific growth conditions.

Here we carried out a three factor design to dissect the role of growth rate on the transcriptional program of yeast. The three factors were specific growth rate, nutrient limitation (carbon/nitrogen limitation), and oxygen availability. For the specific growth rate, multiple levels, i.e. 0.03, 0.1 and 0.2 h^-1 ^were evaluated. In the context of growth rate studies, the effect of oxygen availability has not yet been considered. Beyond identifying growth rate dependent genes independent of nutrient limitation and oxygen availability, we sought to use recently developed systems biology tools to distinguish transcription factors (TFs) that may coordinate and regulate the processes that control cellular growth (e.g. cell cycle period, protein biosynthesis, and energy metabolism).

## Results and discussion

### A three-factor design to investigate growth rate dependent genes

**Figure 1:**
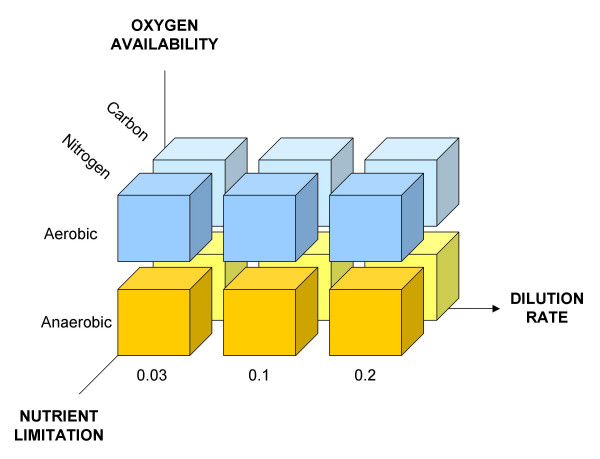
**Experimental design**. Each block represents one of the 12 possible combinations among the three experimental factors (oxygen availability, nutrient limitation and dilution rate). Each experiment was carried out in triplicate, therefore a total of 36 different cultivations were performed. Dilution rate values are given in h^-1^.

To study the growth-rate related transcriptional response in *S. cerevisiae *CEN.PK113-7D, we applied a systems approach that integrated transcriptome measurements with data from protein-DNA interaction networks. A 2 × 2 × 3 factorial design was pursued resulting in 12 different growth conditions (Fig. 1), which have been investigated in triplicate. Specifically, steady-state conditions were chosen to perturb (a) specific growth rate (equal to the dilution rate *D*), (b) nutrient limitation, and (c) oxygen availability. Each factor comprised at least two levels: (a) *D *= 0.03/0.1/0.2 h^-1^, (b) carbon/nitrogen limitation, and (c) aerobiosis/anaerobiosis. Because the specific growth rate (μ) equals the dilution rate (*D*) in our chemostat experiments, the selected range covers cell doubling time (T_2_) between 3.5 and 23.1 h (T_2 _= ln(2)/μ).

**Figure 2:**
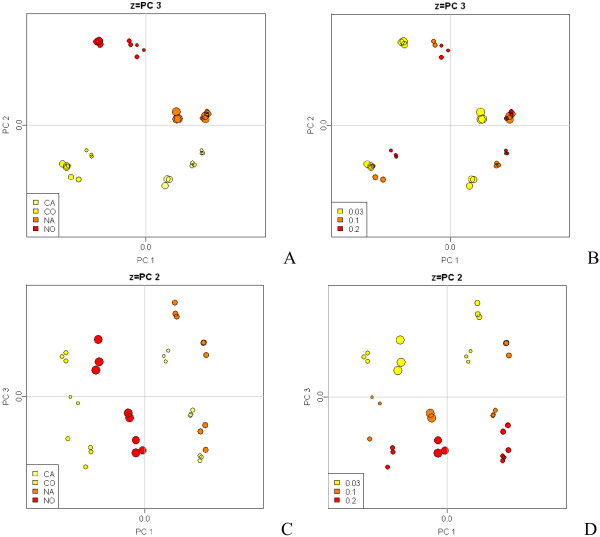
**PCA projection of samples in the first 3 PC dimensions**. Plots A and B show PC dimensions 1 versus 2 as the x- and y-axis and spot size as PC3 in the z-axis. Plots C and D show PC1 vs PC3 and highlight the segregation due to the dilution rate factor in PC3. Color in A and C represents a different combination of these two factors: NO, aerobic nitrogen-limited culture; CO, aerobic carbon-limited culture; NA, anaerobic nitrogen-limited culture; CA, anaerobic carbon-limited culture. Colors in B and D show the dilution rates 0.03, 0.1 and 0.2 h^-1^.

We first collected genome-wide transcription profiles from each steady-state using the Affymetrix GeneChip platform. To reduce data dimensionality and explore the data structure, Principal Components Analysis (PCA) was applied to the normalized microarray data (Fig. 2). Three main principal components were observed, comprising 69% of the variance (see Additional file 1). Strikingly, the PCA projections revealed that the three main principal components segregate the data along the three factors of our factorial design. The factor giving the greatest variance was oxygen availability (O-A split along PC1). The second largest source of variability was observed for nutrient limitation (C-N split along PC2), followed by dilution rate (growth rate split along PC3; Fig. 2A–C). While PC1 shows a clear separation between aerobic and anaerobic conditions, PC2 only distinctly separates the carbon and nitrogen limited conditions for the aerobic case. This is probably due to the fact that in the absence of oxygen only fermentative metabolism is possible, while both respirofermentative (N-limitation) and fully respiratory (C-limitation) metabolism may occur in aerobic conditions. The third factor, specific growth rate, also shows good groupings, although not as distinct as for the other factors (Fig. 2B–D). This is consistent with the transcriptome data from Castrillo *et al*. [13], in which C-limited cultivations were strongly segregated from the other nutrient limited conditions. Notably, the high reproducibility of the replicates demonstrates the quality of our data.

### Functional analysis of the 268 growth rate dependent genes

**Figure 3:**
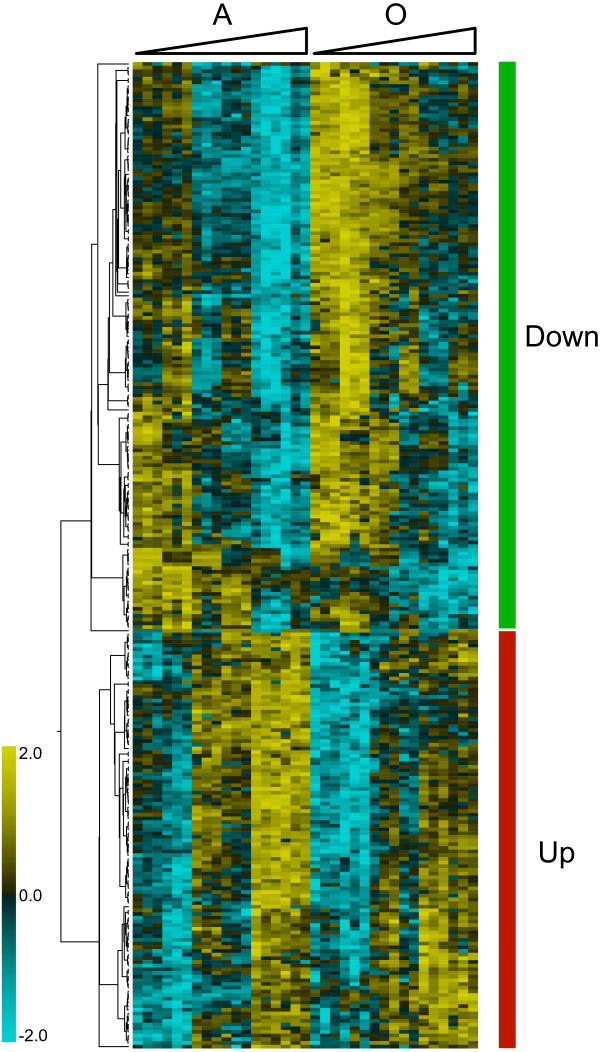
**Hierarchical clustering of growth rate dependent genes**. The columns represent the experiments and the left hand side of the cluster refers to the anaerobic (A) dataset, while the right hand side to the aerobic (O) dataset. The columns are ordered at increasing dilution rate values (0.03 - 0.1 - 0.2 h^-1^), as indicated by the triangles at the top of the clusters. The rows represent the 268 growth rate dependent genes and the two main clusters of up- and down-regulated genes with increasing *D *are shown. The scale of the color bar is based on *z*-score.

To quantitatively reveal which genes had significantly changed expression, MicroArray Analysis of Variance (MAANOVA) was carried out by using mixed-model and Fs test (see Methods and Additional file 2). This test permitted the discovery of genes showing significant transcriptional changes with respect to each considered factor (specific growth rate, nutrient limitation and oxygen availability). Table 2 shows the number of differently expressed genes for each of the three factors at different cut-off *q*-values. At a false discovery rate (FDR) of 2%, which was selected for further analysis, a total of 268 growth rate dependent genes were identified as significantly changed. To group genes with common expression profiles over the dilution rate range, the selected gene lists were clustered using hierarchical clustering (Fig. 3). Of the 268 significantly changed genes, 114 genes were up-regulated with increasing growth rate and 154 genes were down-regulated with increasing growth rate (see Additional file 3). The significantly changed genes are linearly correlated (either negatively or positively) with increasing growth rate (see Additional file 1). Consistent with the PCA analysis, the factor showing the most prominent segregation was oxygen availability. It is possible that this result, in part, reflects the distribution of experimental effort (see Methods).

**Table 2:** The number of significantly changed genes (MAANOVA analysis) at different *q*-value threshold values

*q-value threshold*	*0.01*	*0.02*	*0.03*	*0.04*	*0.05*
Dilution rate	0	268	494	720	938
Nutrient limitation	373	504	579	642	698
Oxygen availability	1208	1933	2038	2355	2594

To determine significantly enriched Gene Ontology (GO) process terms within the up-regulated and down-regulated growth rate dependent gene clusters, we used the *Saccharomyces *Genome Database (SGD)-GO tools (significance at P ≤ 0.01; see Additional file 3). Among genes up-regulated with increasing growth rate, biosynthetic processes were the most significantly enriched (Table 3). In particular, genes involved in ribosome biogenesis and assembly, translation, and protein biosynthesis were over-represented. Nearly half of the up-regulated genes (53/114) encoded for components of the ribosome complex. These results suggest that faster growing cells build biomass more efficiently and are consistent with previous reports [12-15].

**Table 3:** GO annotation based on the Biological Process ontology for growth rate dependent genes

*GO Term*	*Gene hits*	*Cluster frequency*	*P-value*
*Up-regulated Genes (114)*			
cellular biosynthetic process	61	53.5%	1.58E-21
translation	51	44.7%	8.27E-21
biosynthetic process	66	57.9%	1.61E-20
macromolecule biosynthetic process	55	48.2%	4.28E-19
cellular protein metabolic process	59	51.8%	3.95E-11
protein metabolic process	60	52.6%	4.11E-11
cellular macromolecule metabolic process	60	52.6%	4.78E-11
primary metabolic process	90	78.9%	7.37E-11
gene expression	62	54.4%	1.29E-10
cellular metabolic process	91	79.8%	4.39E-10
metabolic process	92	80.7%	9.58E-10
cellular process	101	88.6%	8.12E-07
macromolecule metabolic process	77	67.5%	8.86E-07
ribosome biogenesis and assembly	21	18.4%	4.20E-04
ribosomal subunit assembly	8	7.0%	6.90E-04
ribosome assembly	8	7.0%	2.82E-03
ribonucleoprotein complex biogenesis and assembly	21	18.4%	5.59E-03
			
*Down-regulated Genes (154)*			
cellular carbohydrate metabolic process	18	11.8%	2.90E-04
carbohydrate metabolic process	18	11.8%	1.14E-03
macromolecule catabolic process	22	14.4%	1.86E-03
response to stress	26	17.0%	6.33E-03
catabolic process	24	15.7%	9.30E-03
energy reserve metabolic process	7	4.6%	9.34E-03

Gene hits indicate the number of genes in the clusters of up-/down-regulated genes belonging to that particular GO term; the value is also given as percentage (cluster frequency). *P*-values are provided as a score of significance (cut-off ≤ 0.01).

Among the 154 down-regulated genes, the most over-represented GO terms were response to stress, carbohydrate metabolic process, and catabolic process (Table 3). More specifically, genes encoding proteins involved in ER associated protein catabolism (*HRD3*), vacuole homeostasis (*FAB1*, *GGA1*), ubiquitin cycle (*APC9*, *RTT101*, *UBC8*) and ubiquitin-dependent protein catabolism (*MET30*, *RPN4*, *RPN14*, *YFL006W*) show lower expression levels at higher specific growth rates. *RPN4*, for example, regulates cellular levels of the proteasome [16,17]. While gene expression required for protein synthesis increases with increasing growth rate, gene expression required for protein degradation decreases. It is tempting to speculate that increased protein degradation processes at lower growth rates, typically under sub-optimal conditions, is a survival mechanism designed to more efficiently re-use possible resources.

Strikingly, 11% of down-regulated genes have kinase activity (only 2.8% of yeast genes have kinase activity according to SGD), suggesting a possible role for phosphorylation in regulating the growth rate response. In addition, down-regulated genes having an unknown biological process (22.7%) or function (35.1%) were over-represented. The lack of annotation may be a result of these genes being expressed weakly under the rapid growth conditions used in most microarray experiments [12].

To identify metabolites in yeast around which mRNA expression changes are significantly associated, we applied the Reporter Metabolite Algorithm [18] (see Methods). The most significant Reporter Metabolites are listed in Table 4. These metabolites participate in diverse metabolic pathways from nucleotide and amino acid metabolism, to phospholipid synthesis and the pentose phosphate pathway. Orotate, for example, is involved in the *de novo *synthesis of pyrimidine ribonucleotides. A closer look revealed that *URA5*, whose gene product catalyzes orotate phosphoribosyl transferase, was among the significantly up-regulated genes with increasing growth rate. *URA5 *is not regulated by pathway intermediates and our analysis suggests that transcriptional control of this critical enzyme involved in DNA synthesis helps to mobilize resources necessary for growth. It is striking that ATP, which participates in more reactions than any other metabolite [19], is among the most significant Reporter Metabolites. This result suggests that gene expression of enzymes involved in ATP production and consumption reactions is significantly regulated over changes in specific growth rate. In summary, the Reporter Metabolite results highlight the broad impact that growth rate has across metabolism.

**Table 4:** Reporter Metabolite analysis

*Reporter Metabolites*	*Number of neighbors*	*P-value*
Orotate	3	7.10E-04
D-Mannose 6-phosphate	5	9.71E-04
Spermidine	3	1.68E-03
alpha, alpha-Trehalose	4	3.30E-03
5-Phospho-alpha-D-ribose 1-diphosphate	17	5.15E-03
1-(5'-Phosphoribosyl)-5-amino-4-imidazolecarboxamide	4	5.22E-03
D-Ribose 5-phosphate	18	7.42E-03
Dolichyl beta-D-mannosyl phosphate	7	7.60E-03
FAD	2	9.45E-03
1-Phosphatidyl-D-myo-inositol 4,5-bisphosphate	3	9.99E-03
beta-D-Glucose	3	1.00E-02
ATP	113	1.02E-02
5'-Methylthioadenosine	2	1.05E-02
alpha-D-Glucose 6-phosphate	11	1.19E-02
O-Phospho-4-hydroxy-L-threonine	2	1.26E-02
N6-(L-1,3-Dicarboxypropyl)-L-lysine	2	1.37E-02
Glycogen	4	1.42E-02
Urea-1-carboxylate	1	1.69E-02
(S)-Dihydroorotate	2	1.75E-02
2-Phenylacetamide	1	1.82E-02
Phenylacetic acid	1	1.82E-02
Indole-3-acetamide	1	1.82E-02
Indole-3-acetate	1	1.82E-02
(S)-1-Pyrroline-5-carboxylate	1	1.82E-02
L-1-Pyrroline-3-hydroxy-5-carboxylate	1	1.82E-02
trans-4-Hydroxy-L-proline	1	1.82E-02

Reporter Metabolite analysis [18] identifies metabolites around which the most significant transcriptional changes occur. The number of neighbors indicates the number of genes whose products catalyze a reaction involving that particular metabolite. The algorithm took as input the MAANOVA analysis referring to dilution rate effect. The *P*-value gives a measure of significance and all results < 0.02 are reported.

### Transcription factor control of growth rate dependent genes

**Figure 4:**
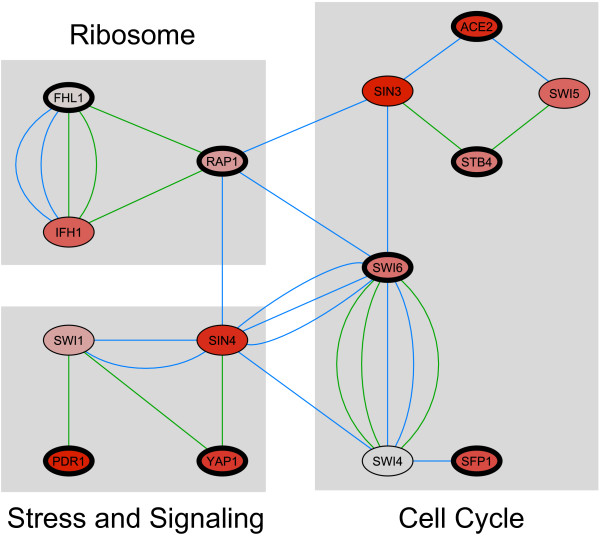
**Network of TFs regulating the genes up-regulated with increasing dilution rate**. Nodes with thicker outlines contain the TFs found in our analysis (YAP5, YAP6, SMP1, GAT3 and BAS1 do not map into this network). The connectivity among nodes is based on the interactions stored at BioGRID database [58] and the interaction types can be divided in two groups: (1) genetic interactions, which can be detected by dosage rescue, synthetic rescue, synthetic growth defect, synthetic lethality, phenotypic enhancement and phenotypic suppression (blue edges); and (2) protein interaction, detected by affinity capture-MS, affinity capture-western, reconstituted complex and two-hybrid (green edges). See Additional file 3 for details about these interactions. Moreover, nodes are colored according to the expression levels of the genes encoding the TFs and a grey-red scale is used (red color indicates higher expression levels). In this network, the TF expression values from experiments at 0.2 h^-1 ^are depicted. No significant differences in TF expression values were observed at different dilution rates (see Additional file 1). The network was drawn by using Cytoscape [59].

To identify and score TFs that might regulate the processes that control cell growth, we scored the significance of overlap between the 268 growth rate dependent genes and known TF target sets [20,21] (Table 5, hypergeometric test at P < 0.01). In total, this analysis revealed 13 TFs having significantly enriched target sets (Fig. 4) for genes up-regulated with increasing growth rate. Fhl1, Rap1, Sfp1, and Yap5 are involved in regulating ribosomal protein gene expression. Ace2 and Swi6 participate in cell cycle regulation. Yap1, Yap6, Smp1, and Pdr1 are involved in stress response and signaling. Bas1 is involved in amino acid and nucleotide biosynthesis, while Stb4 and Gat3 have unclear roles. The connectivity of TFs with enriched targets demonstrates how the global response of growth rate dependent genes may be controlled (Fig. 4). Sin4, Rap1, Swi6, and Swi4 appear to coordinate the response by linking protein synthesis, the cell cycle, and the stress response. No significant TFs were found when the same TF analysis was performed for the down-regulated genes.

**Table 5:** Transcription factor target set enrichment results

*TFs*	*Log10(p-value)*	*Overlap*	*Set1*	*Set2*	*Background*
Harbison *et al*. (YPD), p < 0.01					
FHL1	-28.44	42	114	213	5636
RAP1	-16.52	42	114	414	5636
GAT3	-9.68	23	114	179	5636
SMP1	-4.45	17	114	180	5636
YAP5	-4.1	16	114	168	5636
PDR1	-3.48	15	114	164	5636
					
Harbison *et al*. (Other), p < 0.01					
FHL1 (rapa)	-27.96	42	114	220	5636
FHL1 (sm)	-24.83	44	114	294	5636
FHL1 (H_2_O_2_-Hi)	-16.35	30	114	189	5636
RAP1 (sm)	-13.11	37	114	392	5636
SFP1 (sm)	-8.44	18	114	118	5636
					
Beyer *et al*. SLL > 4					
FHL1	-27.43	51	114	379	5636
RAP1	-20.29	34	114	196	5636
SFP1	-18.9	28	114	129	5636
STB4	-17.91	29	114	153	5636
SWI6	-16	42	114	430	5636
YAP6	-15.16	32	114	242	5636
YAP1	-14.47	35	114	314	5636
ACE2	-10.97	32	114	335	5636
BAS1	-10.64	22	114	147	5636

Target sets defined by Harbison *et al*. [21] chIP-chip study. *p*-values < 0.01 for YPD and other growth conditions are indicated (rapa: rapamycin, sm: sulfometuron methyl, H_2_O_2_-Hi, hydrogen peroxide 4 mM). Sets were also analyzed for Beyer *et al*. [20] derived target sets using sum of log-likelihood (SSL) > 4.

Fhl1, Rap1, and Sfp1 were the TFs with the greatest enrichment of growth-rate dependent target genes (Table 5). These TFs are all involved in ribosomal protein (RP) gene transcription. There are 138 RP genes in yeast, and their expression accounts for more than 50% of the RNA pol II dependent transcription [22]. Rap1 participates in ribosomal gene expression [23-25] and is involved in moving nucleosomes from a certain region of chromatin in order to allow Fhl1 and Ifh1 to trigger RP gene transcription [26-29].

Ace2 and Swi6 are known cell cycle regulators [30] and our TF enrichment analysis suggests a role for these two TFs in controlling growth rate, which remains a hypothesis. Swi6 is part of the two heterodimeric transcriptional regulators SBF (Swi4/Swi6) and MBF (Mbp1/Swi6) [31], that act in the early cell cycle (G1 phase). Ace2, instead, plays an important role during the M phase. Previously, the effect of Ace2 on the length of G1 phase has been reported by Laabs *et al*. [32], who demonstrated that a G1 specific delay in yeast daughter cells is due to this TF. Little is known about Stb4 (SGD classifies Stb4 as having an unknown biological process): it binds to Swi5 [33] and a two-hybrid screen [34] found that it binds to Sin3. We hypothesize that identification of Stb4 as a principal regulating TF in our study, and the close association of it with Swi6 and Ace 2 (Fig. 4), may hint at a possible role for Stb4 in regulating the cell cycle.

Highlighting the importance of both protein biosynthesis and cell cycle progression in controlling growth rate, Sfp1 was also identified in the TF enrichment analysis. Jorgensen *et al*. [35] suggested that Sfp1 activates RP gene transcription by influencing the nuclear localization of Fhl1 and Ifh1. The TOR and PKA pathways, previously identified [13] as critical in controlling growth rate, participate in keeping Sfp1 in the nucleus [36]. Sfp1 also modulates cell cycle progression in the late G1 phase (Start) by controlling cell size in eukaryotic cells [37,38]. Cell cycle progression in the late G1 phase (Start) is dependent on the attainment of a critical cell size and critical translation rate [39].

Several identified TFs with significantly enriched targets are primarily involved in the stress response. Yap1 regulates the expression of oxidative stress response genes [40]. Chua *et al*. [41] have indicated that Yap1 overexpression induces genes involved in translation and tRNA metabolism. Yap6 is known to have a role in salt tolerance [42] and recently Steinfeld *et al*. [43] have indicated a role in regulation of sugar transport. Pdr1 is a zinc finger transcription factor whose target genes carry out ABC transport, other transport, and membrane lipid and cell wall biosyntheses [44]. We have previously proposed a role for Pdr1 in DNA damage response process and showed that Yap5 and Swi5 targets overlap significantly with Pdr1 targets in absence of the damaging agent [45].

In summary, the Reporter Metabolite and TF enrichment analyses both support the conclusion that in yeast changes in growth rates are associated with the regulation of protein synthesis, the cell cycle, and the stress response. For example, four TFs involved in regulation of protein synthesis genes are identified. In agreement, the Reporter Metabolite analysis identifies ATP. Thus, genes encoding products that catalyze reactions involving ATP, and one of the most energy intensive processes of the cell, are observed as being significantly changed. In addition, identification of cell cycle regulators is consistent with results from Reporter Metabolite analysis suggesting that regulation of metabolic pathways of DNA synthesis (the *de novo *synthesis of pyrimidine ribonucleotides) have significant transcriptional changes.

### Comparison with previous growth rate studies

Compared with earlier studies on the influence of the specific growth rate on global transcription, our analysis provides a much more moderate estimate of the number of growth rate dependent genes. This is likely due to two main reasons. First, the three-factor design employed here de-emphasizes genes that might be significant when oxygen availability is not considered. Second, the statistical methods and significance thresholds among the studies are different. Our previous study [12], for example, found the largest number of growth rate dependent genes (~2400). However, that study used a newly developed consensus clustering algorithm to group similar genes that correlated with growth rate [46]. As another illustration, Castrillo *et al*. [13] identified about 900 growth rate dependent genes by performing analysis of covariance (ANCOVA) and applying a *q*-value threshold of ≤ 0.05 for significance. At this threshold, their results are consistent with our findings (978 genes, *q*-value ≤ 0.05; see Table 2). The number of genes specifically overlapping between the work of Castrillo *et al*. and this study at a *q*-value threshold of 0.05 is 315. Using our more conservative cut-off, the overlap is 119.

Given differences between experimental designs and approaches for determining growth rate dependent genes, it is perhaps not surprising that few common genes are observed among our results and the three previous studies (see Additional file 1). Specifically, 21 up-regulated genes and 10 down-regulated genes were shared (Table 6 and 7). Among the common up-regulated genes, 11 were involved in translation (mostly RP genes) and 3 in sphingolipid biosynthesis (*FEN1*, *SUR4*, *URA7*). Of common down-regulated genes, 3 had unknown process (*YDR262W*, *YMR090W*, *YOL153C*) and 4 were involved in regulation of the enzyme fructose-1,6-bisphosphatase, Fbp1 (*PFK26*, *VID28*, *VID30*, *YLR345W*). Despite only a small overlap of specific genes among studies, significantly enriched GO Biological Process terms identified the same overarching biological changes. Considering the substantial variation between the different studies, our multi-factorial dataset is valuable for obtaining robust answers from queries on the effect of growth on transcription of different genes. Due to our multi-factorial design, our dataset is also valuable for evaluation of e.g. the effect of nutritional state independent of growth rate and oxygen availability.

**Table 6:** Common up-regulated genes among growth rate studies

*ORF*	*Gene Name*	*Description*
YBL039C	URA7	Major CTP synthase isozyme (see also URA8), catalyzes the ATP-dependent transfer of the amide nitrogen from glutamine to UTP, forming CTP, the final step in de novo biosynthesis of pyrimidines; involved in phospholipid biosynthesis
YBR189W	RPS9B	Protein component of the small (40S) ribosomal subunit; nearly identical to Rps9Ap and has similarity to E. coli S4 and rat S9 ribosomal proteins
YBR191W	RPL21A	Protein component of the large (60S) ribosomal subunit, nearly identical to Rpl21Bp and has similarity to rat L21 ribosomal protein
YCR034W	FEN1	Fatty acid elongase, involved in sphingolipid biosynthesis; acts on fatty acids of up to 24 carbons in length; mutations have regulatory effects on 1,3-beta-glucan synthase, vacuolar ATPase, and the secretory pathway
YDL083C	RPS16B	Protein component of the small (40S) ribosomal subunit; identical to Rps16Ap and has similarity to E. coli S9 and rat S16 ribosomal proteins
YDR064W	RPS13	Protein component of the small (40S) ribosomal subunit; has similarity to E. coli S15 and rat S13 ribosomal proteins
YDR144C	MKC7	GPI-anchored aspartyl protease (yapsin) involved in protein processing; shares functions with Yap3p and Kex2p
YDR321W	ASP1	Cytosolic L-asparaginase, involved in asparagine catabolism
YEL040W	UTR2	Cell wall protein that functions in the transfer of chitin to beta(1-6)glucan; putative chitin transglycosidase; glycosylphosphatidylinositol (GPI)-anchored protein localized to the bud neck; has a role in cell wall maintenance
YER009W	NTF2	Nuclear envelope protein, interacts with GDP-bound Gsp1p and with proteins of the nuclear pore to transport Gsp1p into the nucleus where it is an essential player in nucleocytoplasmic transport
YGL076C	RPL7A	Protein component of the large (60S) ribosomal subunit, nearly identical to Rpl7Bp and has similarity to E. coli L30 and rat L7 ribosomal proteins; contains a conserved C-terminal Nucleic acid Binding Domain (NDB2)
YKL081W	TEF4	Translation elongation factor EF-1 gamma
YLR186W	EMG1	Protein required for the maturation of the 18S rRNA and for 40S ribosome production; associated with spindle/microtubules; nuclear localization depends on physical interaction with Nop14p; may bind snoRNAs
YLR325C	RPL38	Protein component of the large (60S) ribosomal subunit, has similarity to rat L38 ribosomal protein
YLR372W	SUR4	Elongase, involved in fatty acid and sphingolipid biosynthesis; synthesizes very long chain 20-26-carbon fatty acids from C18-CoA primers; involved in regulation of sphingolipid biosynthesis
YML036W	CGI121	Protein involved in telomere uncapping and elongation as component of the KEOPS protein complex with Bud32p, Kae1p, Pcc1p, and Gon7p; also shown to be a component of the EKC protein complex; homolog of human CGI-121
YML063W	RPS1B	Ribosomal protein 10 (rp10) of the small (40S) subunit; nearly identical to Rps1Ap and has similarity to rat S3a ribosomal protein
YMR318C	ADH6	NADPH-dependent medium chain alcohol dehydrogenase with broad substrate specificity; member of the cinnamyl family of alcohol dehydrogenases; may be involved in fusel alcohol synthesis or in aldehyde tolerance
YOL040C	RPS15	Protein component of the small (40S) ribosomal subunit; has similarity to E. coli S19 and rat S15 ribosomal proteins
YOL120C	RPL18A	Protein component of the large (60S) ribosomal subunit, identical to Rpl18Bp and has similarity to rat L18 ribosomal protein; intron of RPL18A pre-mRNA forms stem-loop structures that are a target for Rnt1p cleavage leading to degradation
YPL144W	YPL144W	Putative protein of unknown function; green fluorescent protein (GFP)-fusion protein localizes to the cytoplasm; null mutant is viable, exhibits shortened telomeres

**Table 7:** Common down-regulated genes among growth rate studies

*ORF*	*Gene Name*	*Description*
YOL153C	YOL153C	Hypothetical protein
YLR345W	YLR345W	Similar to 6-phosphofructo-2-kinase/fructose-2,6-bisphosphatase enzymes responsible for the metabolism of fructoso-2,6-bisphosphate; mRNA expression is repressed by the Rfx1p-Tup1p-Ssn6p repressor complex; YLR345W is not an essential gene
YGR070W	ROM1	GDP/GTP exchange protein (GEP) for Rho1p; mutations are synthetically lethal with mutations in rom2, which also encodes a GEP
YMR090W	YMR090W	Putative protein of unknown function with similarity to DTDP-glucose 4,6-dehydratases; green fluorescent protein (GFP)-fusion protein localizes to the cytoplasm; YMR090W is not an essential gene
YDR262W	YDR262W	Putative protein of unknown function; green fluorescent protein (GFP)-fusion protein localizes to the vacuole and is induced in response to the DNA-damaging agent MMS; gene expression increases in response to Zymoliase treatment
YGL121C	GPG1	Proposed gamma subunit of the heterotrimeric G protein that interacts with the receptor Grp1p; involved in regulation of pseudohyphal growth; requires Gpb1p or Gpb2p to interact with Gpa2p
YIL107C	PFK26	6-phosphofructo-2-kinase, inhibited by phosphoenolpyruvate and sn-glycerol 3-phosphate, has negligible fructose-2,6-bisphosphatase activity, transcriptional regulation involves protein kinase A
YGR087C	PDC6	Minor isoform of pyruvate decarboxylase, key enzyme in alcoholic fermentation, decarboxylates pyruvate to acetaldehyde, regulation is glucose- and ethanol-dependent, involved in amino acid catabolism
YIL017C	VID28	Protein involved in proteasome-dependent catabolite degradation of fructose-1,6-bisphosphatase (FBPase); localized to the nucleus and the cytoplasm
YGL227W	VID30	Protein involved in proteasome-dependent catabolite degradation of fructose-1,6-bisphosphatase (FBPase); shifts the balance of nitrogen metabolism toward the production of glutamate; localized to the nucleus and the cytoplasm

## Conclusion

By increasing the number of experimental factors, we have identified a more conservative set of growth-rate dependent genes. Specifically, our analysis has identified 268 specific growth rate dependent genes. Results of a gene function analysis were found to be in agreement with previous studies [12-14]. New insight into the regulation of growth rate regulated genes has also been provided. Specifically, 13 TFs have been identified as related to genes whose transcripts level increased with increasing growth rate and 8 of these are connected in a map of regulatory pathways supported by known protein-DNA interactions. Supported by the Reporter Metabolite analysis, the TFs that coordinate growth rate dependent genes are primarily involved in protein synthesis, the cell cycle, and the stress response. Strikingly, down-regulated genes with increasing growth rate did not show common regulation, likely due to the high percentage of uncharacterized genes. We have shown that multi-factor designs, combined with a systems biology approach, can enhance our knowledge about yeast responses to growth rate. This approach will be valuable for studying any other environmental or genetic factor of interest.

## Methods

### Strain and chemostat cultivations

The reference laboratory strain *S. cerevisiae *CEN.PK113-7D (*MAT***a**) [47] was grown in well controlled 2 liter jacketed chemostats (Braun Biotech and Applikon) with a constant working volume of 1.0 liter. Cultivations were carried out (in triplicates) in aerobic/anaerobic and carbon/nitrogen limited conditions, at 30°C with a stirrer speed of 800 rpm, pH of 5.0 (maintained by automatic addition of 2 N potassium hydroxide) and dilution rates of 0.03, 0.1 and 0.2 h^-1^. Aerobic conditions were maintained by sparging the cultures with air (1.0 L min^-1^) and the concentration of dissolved oxygen was measured with Mettler Toledo polarographic electrode. Anaerobic conditions were maintained by sparging the medium reservoir and the fermentor with pure nitrogen gas (0.5 L min^-1^). Moreover, oxygen diffusion was minimized by using norprene tubing and butyl septa. The bioreactors were fitted with cooled condensers (2 – 4°C) and the off-gas was led to a gas analyzer (INNOVA and NGA 2000 Rosemount) to measure the content of CO_2 _and O_2_. Steady-state was reached when at least five residence times had passed since starting the continuous cultivation and carbon dioxide evolution, dry weight measurements, and HPLC measurements of extracellular metabolites were constant.

The experimental work was divided into two efforts. Aerobic cultivations were carried out in the laboratory of Jens Nielsen. Anaerobic cultivations were carried out in the laboratory of Jack T. Pronk. Considerable effort was invested in standardizing the strain, growth conditions, sampling protocols, and analytical procedures. Our groups previously published a report that concluded that microarray experiments in our laboratories were excellently comparable [7]. Triplicate cultivations were carried out for each set of conditions to reduce bias that might unexpectedly arise and to account for biological variance.

### Media

The medium composition was as previously described by Tai *et al*. [8]. For N-limited cultivations, residual glucose concentration in the chemostat was targeted to 17 ± 2 g L^-1^. This was to sustain glucose repression at the same level in all cultivations. The mineral medium composition for the N-limited cultivations was (amounts per liter): (NH_4_)_2_SO_4 _1 g, KH_2_PO_4 _3 g, K_2_SO_4 _5.3 g, MgSO_4_·7H_2_O 0.5 g, Trace Metal Solution 1 mL, antifoaming agent 0.05 mL and vitamin solution 1 mL. The mineral medium composition for the C-limited cultivations was (amounts per liter): (NH_4_)_2_SO_4 _5 g, KH_2_PO_4 _3 g, MgSO_4_·7H_2_O 0.5 g, Trace Metal Solution 1 mL, antifoaming agent 0.05 mL and vitamin solution 1 mL. The inlet glucose concentration was ca. 11 and 25 g L^-1 ^for aerobic and anaerobic experiments, respectively. Moreover, anaerobic cultivation medium was supplemented with Tween 80/ergosterol solution (1.25 mL/L).

### Analytical methods

The concentration of biomass at steady-state was determined on a dry weight basis by filtering 5 mL of culture through a pre-weighed 0.45 μm nitrocellulose filter (Gelman Sciences, Ann Arbor, MI). The filter was washed with distilled water, dried in a microwave oven at 150 W for 15 minutes and finally weighed to determine its increase in dry weight. Culture samples (10 mL) for determination of extracellular glucose, succinate, glycerol, acetate, ethanol and pyruvate concentrations were immediately filtered through a 0.2 μm filter (Osmonics, Minnetonka, MN, USA) and the filtrate was stored at -20°C for further analysis. The metabolite concentrations were determined by high pressure liquid chromatography using an Aminex HPX87H column (Biorad) kept at 65°C and eluted at 0.6 mL per minute with H_2_SO_4_. Pyruvate was detected spectrophotometrically by a Waters 486 Tunable Absorbance Detector at 210 nm. Glucose, succinate, glycerol, acetate and ethanol were detected by a Waters 410 Differential Refractometer.

### RNA sampling and isolation

Samples for RNA isolation from aerobic cultivations were taken by rapidly sampling 20 mL of culture into a tube with 35–40 mL of crushed ice in order to decrease the sample temperature to below 2°C in less than 10 seconds. Cells were then centrifuged (4500 rpm at 0°C for 3 minutes), instantly frozen in liquid nitrogen and stored at -80°C until further use. Sampling for RNA isolations from anaerobic cultivations was performed as described by Piper *et al*. [7].

Total RNA was extracted using FastRNA Pro RED kit (QBiogene, Inc, USA) according to manufacturer's instructions after partially thawing the samples on ice. RNA sample integrity and quality was assessed prior to hybridization with an Agilent 2100 Bioanalyzer and RNA 6000 Nano LabChip kit.

### Probe preparation and hybridization to arrays

Messenger RNA extraction, cDNA synthesis and labeling, as well as array hybridization to Affymetrix Yeast Genome S98 arrays, were performed as described in the Affymetrix users' manual [48]. Washing and staining of arrays were performed using the GeneChip Fluidics Station 450 and scanning with the Affymetrix GeneArray Scanner.

### Microarray gene transcription analysis

Affymetrix Microarray Suite v5.0 was used to generate CEL files of the scanned DNA microarrays. These CEL files were preprocessed by using gcrma and affy packages [49,50] available in Bioconductor. Raw data was background corrected by using gcrma package and normalized by using qspline method [51]. Probe summarization was made using only the perfect match (PM) values and median polish settings [52].

Principal Components Analysis (PCA) was performed in order to elucidate the relative importance of the three factors characterizing our experimental design: oxygen availability, nutrient limitation and dilution rate. To select genes whose expression levels were related to these factors, MicroArray Analysis of Variance (MAANOVA) was performed with a mixed model ANOVA with the fixed factors 'oxygen', 'nutrient' and 'dilution rate' and a single random factor, 'sample', representing the biological replicates [53]. Among the various F-tests, the so called Fs was chosen [54] and the *q-*value method was used to correct for multiple testing [55], which was shown to be less conservative than the FDR methodology described by Benjamini & Hochberg [56]. The threshold of significance was set at 0.02 for a false discovery rate of 2%. MAANOVA is available as a package in Bioconductor and details of the code are given in Additional file 2. Subsequently, in order to group genes with common expression profiles over the dilution rate range, the selected gene lists were clustered using hierarchical clustering (unweighted pair-group average with a non-centric Pearson correlation based distance) and the Gene Ontology of the generated clusters was investigated [57].

### Reporter Metabolite analysis

Using the entire gene expression data set, we applied the Reporter Metabolite Algorithm [18] with a newly reported genome-scale metabolic model of yeast (Nookaew *et al*., submitted). More specifically, the genome-scale model was converted to a bipartite undirected graph. In this graph, each metabolite node has as neighbors the enzymes catalyzing the formation and consumption of the metabolite. The transcriptome data were mapped on the enzyme nodes using the significant values of gene expression. The normal cumulative distribution function was used to convert the *p*-values to a Z-score. Each metabolite was assigned the average score of its *k *neighboring enzymes, and this score was then corrected for the background by subtracting the mean and dividing by the standard deviation of average scores of 10,000 enzyme groups of size *k *selected from the same data set. These corrected scores were then converted back to *P *values by using the normal cumulative distribution function and the most significant metabolites, Reporter Metabolites, were ranked.

### Transcription factor enrichment analysis

For the genes that were found to be differentially transcribed due to growth rate, we investigated if the set of up- and/or down-regulated genes were enriched for regulation by specific transcription factors. Definitions of transcription factor target sets (protein-DNA interactions) were derived from two different data sources [20,21] at *p*-value threshold 0.01 for the Harbison *et al*. study and sum of log-likelihood threshold 4 for the Beyer *et al*. study. The hypergeometric test was performed for each TF in each of these 2 set definitions versus the up- and down-regulated genes and the resulting *p*-values were Bonferroni adjusted.

## Abbreviations

FDR: False Discovery Rate; GO: Gene Ontology; MAANOVA: MicroArray ANalysis Of Variance; PCA: Principal Components Analysis; RP: Ribosomal Protein; SGD: *Saccharomyces *Genome Database; TF: Transcription Factor.

## Competing interests

The authors declare that they have no competing interests.

## Supplementary Material

Additional file 1 **Supplementary Figures and Tables**. Additional figures and tables about the PCA analysis, consensus cluster analysis and the comparison among the four growth rate studies (Regenberg *et al*., [12]; Castrillo *et al*., [13]; Brauer *et al*., [14]; Fazio *et al*., [present study]). Click here for file

Additional file 2 **Supplementary Methods**. Details of the R code used for the analysis of CEL files. Click here for file

Additional file 3 **Gene Lists, Gene Ontology and TF Interactions**. This *.xls file contains 7 worksheets: (1) full annotation of the 114 (FDR 2%) growth rate-dependent genes up-regulated at increasing dilution rates; (2) full annotation of the 154 (FDR 2%) growth rate-dependent genes down-regulated at increasing dilution rates; (3) full annotation of the nutrient limitation dependent genes (FDR 2%); (4) full annotation of the oxygen availability dependent genes at (FDR 2%); (5–6) Gene Ontology analysis (Process, Function, Component) of the up/down-regulated growth rate genes performed by using GO Term Finder and GO Slim Mapper available at the *Saccharomyces *Genome Database (SGD) website; (7) Detailed description of the interactions of the transcription factor network presented in figure 4 of the paper. Click here for file
