# Supplementary material for: Transcription factor control of growth rate dependent genes in Saccharomyces cerevisiae: A three factor design
Source: BMC Genomics. 2008 Jul 18;9:341. doi: 10.1186/1471-2164-9-341 (PMC2500033; doi:10.1186/1471-2164-9-341)
Supplement: Additional file 1 — Supplementary Figures and Tables. Additional figures and tables about the PCA analysis, consensus cluster analysis and the comparison among the four growth rate studies (Regenberg et al., [12]; Castrillo et al., [13]; Brauer et al., [14]; Fazio et al., [present study]). [file 1471-2164-9-341-S1.doc]

Supplementary Figures and Tables

Transcription factor control of growth rate dependent genes in *Saccharomyces cerevisiae*: a three factor design

Alessandro Fazio, Michael C. Jewett, Pascale Daran-Lapujade, Roberta Mustacchi, Renata Usaite, Jack T. Pronk, Christopher T. Workman, Jens Nielsen

Supplementary Figure 1. Singular values from the singular value decomposition (SVD) approach to principal component analysis of microarray expression data. The majority of the data set variance is accounted for in the top three components (see also Fig. 2 of the paper).


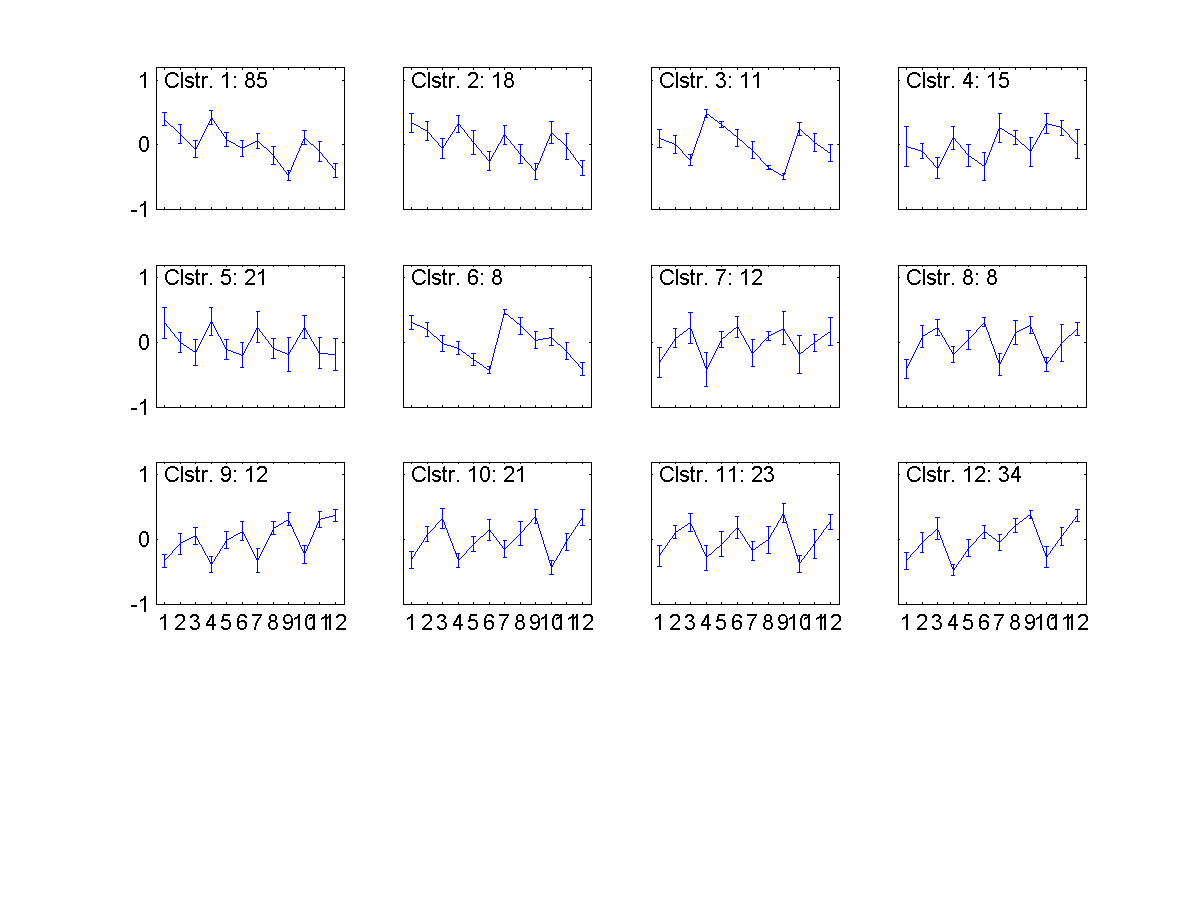


Supplementary Figure 2. The expression values of the 268 growth dependent genes were normalized to a range between -1 and 1, and consensus cluster analysis [1] was performed in order to show the linear correlation between transcripts and increasing dilution rate values. Twelve clusters were obtained. The experimental factor (see Fig. 1 of the paper) are represented, at increasing dilution rate, on the *x*-axis and indicated by numbers: 1-3, aerobic carbon-limited cultures; 4-6, aerobic nitrogen-limited cultures; 7-9, anaerobic carbon-limited cultures; 10-12 anaerobic nitrogen-limited cultures. Moreover, the number of genes belonging to each cluster is shown. Clusters 1 to 6 are characterized by negative slopes, while clusters 7 to 12 refer to genes with positive correlation with dilution rate.

*Up-regulated genes*

**This study**

**Castrillo**

***et al.*, 2007**

**Regenberg**

***et al.*, 2006**

**Brauer**

***et al.*, 2008**

**742**

**1753**

**114**

**493**

**21**

*Down-regulated genes*

**This study**

**Castrillo**

***et al.*, 2007**

**Regenberg**

***et al.*, 2006**

**Brauer**

***et al.*, 2008**

**728**

**672**

**154**

**398**

**10**

Supplementary Figure 3. Comparison of growth rate dependent gene lists among the four considered studies (up-/down-regulation). Numbers refer to significantly changed genes in the indicated study and the two inner circles contain the numbers of common genes.

Supplementary Figure 4. Expression values (log_2_) of the genes encoding for the 13 TFs controlling the up-regulated genes at increasing dilution rates (see Results and Discussion and Figure 4 of the paper). SIN3, IFH1, SWI1, SWI5, SIN4 and SWI4 are also shown. For each gene, expression values relative to the three dilution rates are presented. As it can be seen, changes in dilution rate do not have big impact on the transcription levels of these genes.

Supplementary Table 1. Comparison of growth-rate dependent gene lists. All possible pair-wise combinations among the four studies were considered; numbers indicate the common growth-rate dependent genes (red: up-regulated genes; green: down-regulated genes).

|  | **Fazio *et al.* (present study)** | **Regenberg *et al.* (2006)** | **Castrillo *et al.* (2007)** | **Brauer *et al.* (2008)** |
| --- | --- | --- | --- | --- |
| **Fazio *et al.* (present study)** |  | **66** | **69** | **80** |
| **Regenberg *et al.* (2006)** | **43** |  | **232** | **366** |
| **Castrillo *et al.* (2007)** | **50** | **58** |  | **198** |
| **Brauer *et al.* (2008)** | **82** | **191** | **161** |  |

1. Grotkjaer T, Winther O, Regenberg B, Nielsen J, Hansen LK: **Robust multi-scale clustering of large DNA microarray datasets with the consensus algorithm**. *Bioinformatics* 2006, **22**(1):58-67.

2. Regenberg B, Grotkjaer T, Winther O, Fausboll A, Akesson M, Bro C, Hansen LK, Brunak S, Nielsen J: **Growth-rate regulated genes have profound impact on interpretation of transcriptome profiling in Saccharomyces cerevisiae**. *Genome Biol* 2006, **7**(11):R107.

3. Castrillo JI, Zeef LA, Hoyle DC, Zhang N, Hayes A, Gardner DC, Cornell MJ, Petty J, Hakes L, Wardleworth L *et al*: **Growth control of the eukaryote cell: a systems biology study in yeast**. *J Biol* 2007, **6**(2):4.

4. Brauer MJ, Huttenhower C, Airoldi EM, Rosenstein R, Matese JC, Gresham D, Boer VM, Troyanskaya OG, Botstein D: **Coordination of growth rate, cell cycle, stress response, and metabolic activity in yeast**. *Mol Biol Cell* 2008, **19**(1):352-367.
