# Supplementary material for: Transcription factor control of growth rate dependent genes in Saccharomyces cerevisiae: A three factor design
Source: BMC Genomics. 2008 Jul 18;9:341. doi: 10.1186/1471-2164-9-341 (PMC2500033; doi:10.1186/1471-2164-9-341)
Supplement: Additional file 2 — Supplementary Methods. Details of the R code used for the analysis of CEL files. [file 1471-2164-9-341-S2.pdf]

R CODE USED FOR THE DATA ANALYSIS IN THE PAPER:

Transcription factor control of growth rate dependent genes in *Saccharomyces cerevisiae*: a three factor design

Alessandro Fazio, Michael C. Jewett, Pascale Daran-Lapujade, Roberta Mustacchi, Renata Usaite, Jack T. Pronk, Christopher T. Workman, Jens Nielsen*

April 3, 2008

1 Loading the packages

```
> source("http://bioconductor.org/biocLite.R")
> library(affy)
> library(gcrma)
> library(MASS)
> library(ygs98)
> library(annaffy)
> library(geneplotter)
> library(affyPLM)
> library(RColorBrewer)
> library(maanova)
> library(qvalue)
```

2 Quality Control Assessment

```
> ExpData <- ReadAffy()
> ExpData

> Pset <- fitPLM(ExpData)
> image(Pset)
> image(Pset, type = "resids")

> cols <- brewer.pal(8, "Set1")
> RLE(Pset, col = cols, main = "Relative Log Expression values")
> NUSE(Pset, col = cols, main = "Normalized Unscaled Standard Errors")
```

Due to low quality, data from one of the GeneChip ("NO_D1_1_s98.CEL") was not considered for further analysis.

*Corresponding author (nielsenj@chalmers.se)

3 Data Preprocessing

Raw data was background corrected by using `gcrma` package and normalized by using qspline method (Workman *et al.*, 2002). No adjustment was made on the pm values and medianpolish method was used for probe summarization (Irizarry *et al.*, 2003).

```
> affinity.info.ygs98 <- compute.affinities(cdfName(ExpData))
> ExpData.bgadj <- bg.adjust.gcrma(ExpData, affinity.info = affinity.info.ygs98, type = "affinities")
> ExpSet <- expresso(ExpData.bgadj, bgcorrect.method = "none", normalize.method = "qspline",
+     pmcorrect.method = "pmonly", summary.method = "medianpolish")
```

```
> ExpSet
> MAplot(ExpData, plot.method = "smoothScatter", log = T, type = "pm")
> MAplot(ExpSet, plot.method = "smoothScatter", log = F)
```

4 Extracting SGD Genes

Of the 9335 probesets present on Yeast S98 GeneChip, only the ones with SGD sequence source were considered for subsequent analysis. The IDs of these 7079 probesets were given as input in the "probelist.txt" file.

```
> probelist <- read.table("probelist.txt")
> index <- pmatch(as.character(probelist[, 1]), rownames(exprs(ExpSet)))
> exp.values <- exprs(ExpSet)[index, ]
> dim(exp.values)
> colnames(exp.values)
```

```
> col.names <- c("AFFYID", colnames(exp.values))
> write.table(t(col.names), file = "Normalized_ExprValues.txt", quote = F, sep = "\t", col.names = F,
+     row.names = F)
> write.table(exp.values, file = "Normalized_ExprValues.txt", quote = F, sep = "\t", col.names = F,
+     row.names = T, append = T)
```

5 MicroArray Analysis of Variance (MAANOVA)

```
> outfile1 <- "Maanova_exprData_Dilution.txt"
> outfile2 <- "Maanova_results_Dilution.html"
> outfile3 <- "Maanova_results_Dilution.txt"
```

This analysis takes as inputs a data file (pre-processed probesets) and a design file (see below).

```
> data <- "Normalized_ExprValues.txt"
> designfile <- "designfile.txt"
```

```
> ciao.raw <- read.madata(data, designfile = designfile, header = TRUE, spotflag = FALSE, metarow = 1,
+     metacol = 1, pmt = 2, probeID = 1)
> summary(ciao.raw)
```

The following is the experimental design (A: Anaerobic, O: aerObic, C: C-limitation, N: N-limitation, D03: dilution rate 0.03 h^-1, D1: dilution rate 0.1 h^-1, D2: dilution rate 0.2 h^-1)

	Array	Dye	Oxygen	Nutrient	Dilution	Sample
1	1	1	A	C	D03	1
2	2	1	A	C	D03	1
3	3	1	A	C	D03	1
4	4	1	A	C	D1	2
5	5	1	A	C	D1	2
6	6	1	A	C	D1	2
7	7	1	A	C	D2	3
8	8	1	A	C	D2	3
9	9	1	A	C	D2	3
10	10	1	O	C	D03	4
11	11	1	O	C	D03	4
12	12	1	O	C	D03	4
13	13	1	O	C	D1	5
14	14	1	O	C	D1	5
15	15	1	O	C	D1	5
16	16	1	O	C	D2	6
17	17	1	O	C	D2	6
18	18	1	O	C	D2	6
19	19	1	A	N	D03	7
20	20	1	A	N	D03	7
21	21	1	A	N	D03	7
22	22	1	A	N	D1	8
23	23	1	A	N	D1	8
24	24	1	A	N	D1	8
25	25	1	A	N	D2	9
26	26	1	A	N	D2	9
27	27	1	A	N	D2	9
28	28	1	O	N	D03	10
29	29	1	O	N	D03	10
30	30	1	O	N	D03	10
31	31	1	O	N	D1	11
32	32	1	O	N	D1	11
33	33	1	O	N	D2	12
34	34	1	O	N	D2	12
35	35	1	O	N	D2	12

A mixed model was used to take into account the biological replicates as source of variation (Churchill, 2004).

```
> anova.full.mix <- fitmaanova(ciao.raw, formula = ~Oxygen + Nutrient + Dilution + Sample, random = ~Sample,
+     method = "REML")
> summary(anova.full.mix)
> resiplot(ciao.raw, anova.full.mix)
```

Statistical significance was assessed by using the Fs test (Cui *et al.*, 2005) and the qvalue-based FDR control (Storey and Tibshirani, 2003) (the chosen cut-off value was set to 0.02).

```
> term <- "Dilution"
> test.Factor.mix <- matest(ciao.raw, anova.full.mix, term = term, n.perm = 1, test.method = c(1,
+     1, 0))
> summary(test.Factor.mix)
```

```
> test.Factor.mix <- adjPval(test.Factor.mix, method = "jsFDR")
> summary(test.Factor.mix)
```

```
> idx.Factor.mix <- volcano(test.Factor.mix, threshold = rep(0.02, 3), method = c("fdr", "fdr",
+     "unadj"), title = "Volcano plot", onScreen = T)
> summary(idx.Factor.mix)
```

```
> Fs.lst <- test.Factor.mix$Fs
> chosen.cutoff <- 0.02
> selected <- which(Fs.lst$adjPtab < chosen.cutoff)
> length(selected)
> probeids2 <- ciao.raw$probeID[selected]
> expr.data2 <- read.delim(file = data, as.is = T)[selected, ]
> chosen.adjPvalue <- Fs.lst$adjPtab[selected]
```

```
> index2 <- order(abs(chosen.adjPvalue), decreasing = F)
> probeids <- probeids2[index2]
> expr.data <- expr.data2[index2, ]
> ordered.adjPvalue <- chosen.adjPvalue[index2]
> write.table(expr.data, file = outfile1, quote = F, sep = "\t", col.names = T, row.names = F)
```

6 Annotation of Dilution-Rate Related Genes

The `annaffy` package was used to annotate selected probesets.

```
> aaf.handler()
> anncols <- aaf.handler()[c(1:13)]
> anntable <- aafTableAnn(probeids, "ygs98", anncols)
```

```
> gene.ORF <- as.list(ygs98ORF)
> gene.name <- as.list(ygs98GENENAME)
> gene.alias <- as.list(ygs98ALIAS)
> gene.descript <- as.list(ygs98DESCRIPTION)
> im <- pmatch(probeids, names(gene.name))
> no.names <- which(is.na(gene.name))
> gene.name[no.names] <- gene.ORF[no.names]
> annot <- cbind(as.character(gene.ORF), as.character(gene.name), as.character(gene.alias), as.character(gene.descri
+     ]
> col.names2 <- c("ORF", "GeneName", "Alias", "Full Description")
> ORFtable <- aafTable(ORF = annot[, 1], GeneName = annot[, 2], Alias = annot[, 3], Description = annot[,
+     4], colnames = col.names2, probeids = probeids)
> table <- merge(anntable, ORFtable)
```

```
> pvalue.table <- aafTable(adjP.value = ordered.adjPvalue, signed = TRUE)
> table <- merge(table, pvalue.table)
```

```
> ES <- as.matrix(read.table(outfile1, header = TRUE, sep = "\t", row.names = 1, as.is = TRUE))
> expr.set <- new("ExpressionSet", exprs = ES)
> expr.table <- aafTableInt(expr.set, colnames = names(expr.data)[2:36], probeids = probeids)
> table <- merge(table, expr.table)
```

```
> saveHTML(table, outfile2, title = "Dilution Rate Effect (Mixed model, Fs, q-value, 0.02)")
> saveText(table, outfile3)
```

7 Session Info

```
> sessionInfo()

R version 2.6.0 (2007-10-03)
i386-pc-mingw32

locale:
LC_COLLATE=English_United States.1252;LC_CTYPE=English_United States.1252;LC_MONETARY=English_United States.1252;LC

attached base packages:
 [1] tcltk     splines   tools     stats     graphics  grDevices utils     datasets  methods   base

other attached packages:
 [1] qvalue_1.12.0       maanova_1.8.0       RColorBrewer_1.0-1  affyPLM_1.14.0      geneplotter_1.16.0
 [6] lattice_0.16-5      annotate_1.16.1     xtable_1.5-2        AnnotationDbi_1.0.6 RSQLite_0.6-3
[11] DBI_0.2-4           annaffy_1.10.0      KEGG_2.0.0          GO_2.0.0            ygs98_2.0.1
[16] MASS_7.2-37         gcrma_2.10.0        matchprobes_1.10.0  affy_1.16.0         preprocessCore_1.0.0
[21] affyio_1.6.1        Biobase_1.16.1

loaded via a namespace (and not attached):
[1] grid_2.6.0         KernSmooth_2.22-21
```

References

Churchill, Gary A (2004) Using ANOVA to analyze microarray data. *Biotechniques* **37**: 173–5, 177.

Cui, Xiangqin, Hwang, J. T Gene, Qiu, Jing, Blades, Natalie J, and Churchill, Gary A (2005) Improved statistical tests for differential gene expression by shrinking variance components estimates. *Biostatistics* **6**: 59–75, URL http://dx.doi.org/10.1093/biostatistics/kxh018.

Irizarry, Rafael A, Hobbs, Bridget, Collin, Francois, Beazer-Barclay, Yasmin D, Antonellis, Kristen J, Scherf, Uwe, and Speed, Terence P (2003) Exploration, normalization, and summaries of high density oligonucleotide array probe level data. *Biostatistics* **4**: 249–264, URL http://dx.doi.org/10.1093/biostatistics/4.2.249.

Storey, John D and Tibshirani, Robert (2003) Statistical significance for genomewide studies. *Proc Natl Acad Sci U S A* **100**: 9440–9445, URL http://dx.doi.org/10.1073/pnas.1530509100.

Workman, Christopher, Jensen, Lars Juhl, Jarmer, Hanne, Berka, Randy, Gautier, Laurent, Nielser, Henrik Bjørn, Saxild, Hans-Henrik, Nielsen, Claus, Brunak, Søren, and Knudsen, Steen (2002) A new non-linear normalization method for reducing variability in DNA microarray experiments. *Genome Biol* **3**: research0048.
